# Transcription‐coupled repair – mechanisms of action, regulation, and associated human disorders

**DOI:** 10.1002/1873-3468.15073

**Published:** 2024-12-20

**Authors:** Yuka Nakazawa, Yasuyoshi Oka, Tomoko Matsunaga, Tomoo Ogi

**Affiliations:** ^1^ Department of Genetics Research Institute of Environmental Medicine (RIeM), Nagoya University Furo‐cho, Chikusa‐ku Nagoya 464‐8601 Japan; ^2^ Department of Human Genetics and Molecular Biology Nagoya University Graduate School of Medicine 65, Tsurumai‐cho, Showa‐ku Nagoya 466‐8550 Japan; ^3^ Division of Animal Medical Science, Center for One Medicine Innovative Translational Research (COMIT), Tokai National Higher Education and Research System Nagoya University Nagoya 464‐8601 Japan; ^4^ Division of Molecular Physiology and Dynamics, Institute for Glyco‐core Research (iGCORE) Tokai National Higher Education and Research System Nagoya 464‐8601 Japan

**Keywords:** AMeDS (aplastic anemia, and dwarfism syndrome), Cockayne syndrome (CS), DNA‐protein crosslinks (DPCs), mental retardation, nucleotide excision repair (NER), RNA polymerase II (RNAPII), transcription‐coupled repair (TCR), trichothiodystrophy (TTD), UV‐sensitive syndrome (UVSS), xeroderma pigmentosum (XP)

## Abstract

The transcription‐coupled repair (TCR) pathway resolves transcription‐blocking DNA lesions to maintain cellular function and prevent transcriptional arrest. Stalled RNA polymerase II (RNAPII) triggers repair mechanisms, including RNAPII ubiquitination, which recruit UVSSA and TFIIH. Defects in TCR‐associated genes cause disorders like Cockayne syndrome, UV‐sensitive syndrome, xeroderma pigmentosum, and recently defined AMeDS. TCR safeguards transcription, linking its failure to neurodegeneration and disease phenotypes.

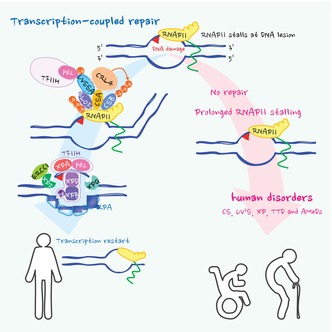

**Abbreviations**
Transcription‐coupled repair: (TCR)
nucleotide excision repair: (NER)
RNA polymerase II: (RNAPII)
Cockayne syndrome: (CS)
aplastic anemia, mental retardation, and dwarfism syndrome: (AMeDS)
Fanconi anemia: (FA)
UV‐sensitive syndrome: (UVSS)
xeroderma pigmentosum: (XP)
trichothiodystrophy: (TTD)
cyclobutane pyrimidine dimer: (CPD)
DNA‐protein crosslink: (DPC)
inter‐strand crosslink: (ICL).

## Review

The intricacies of life rely on the orchestrated expression of a wide variety of genes, a fundamental process for maintaining normal cellular function and the development of our body. At the center of this mechanism lies mRNA transcription, which involves transcribing genetic information encoded in DNA into mRNA molecules by RNA polymerase II (RNAPII). However, this process is frequently fraught with challenges, especially when DNA damage arise and the lesions remain unrepaired [1, 2, 3]. Both external and internal factors, such as environmental toxins and metabolic byproducts, constantly assault the integrity of DNA. When DNA damage occurs on the template strand of actively transcribed genes, it poses a significant obstacle to RNAPII progression. These transcriptional roadblocks, ranging from base adducts to crosslinks and strand breaks, can impede the movement of RNAPII along the DNA template, resulting in the loss of precise transcriptional profiles. These profiles are essential for the timely and accurate expression of gene sets required for proper cellular functions. Consequently, aberrant gene expression may lead to cellular malfunctions and global abnormalities within the organism. To overcome transcriptional stresses, stalled RNAPII complexes activate cellular signal responses, leading to either temporary shutdown of transcription or apoptosis. Therefore, cells employ a specialised mechanism called transcription‐coupled repair (TCR) to resolve cytotoxic transcription‐blocking lesions and resume transcription, thereby maintaining cellular viability [4].

The TCR pathway operates in concert with the transcription machinery to detect and remove DNA lesions specifically from the transcribed DNA strand [5]. Major issues arise when the lesions are compact in size, and the strand with DNA damage encounters RNAPII, causing it to stall and impede forward translocation [6]. Cockayne syndrome (CS) is a rare hereditary progeria that is compromised in handling such situations, caused by mutations in either the *ERCC6/CSB* gene or the *ERCC8/CSA* gene [7, 8]. When RNAPII stalls, the CSB protein ‐an ATP‐dependent chromatin‐remodeling factor‐ plays a crucial role in recruiting the Cullin RING E3 ubiquitin ligase complex 4 (CRL4) along with the RNAPII‐specific adaptor protein CSA [9]. The CSB/CSA‐CRL4 complex facilitates a single DNA damage‐induced ubiquitination of the largest subunit of RNAPII at the RPB1‐Lys1268 residue [10], supported by the interaction between CSA and ELOF1 [11]. It has been demonstrated that compromising the RPB1‐K1268 ubiquitination by introducing a lysine‐to‐arginine single amino acid substitution in mice (*Polr2a* ^K1268R^), combined with a damage‐overloading genetic background, recapitulates major CS phenotypes [10]. This suggests that the RNAPII ubiquitination is a crucial signaling mechanism for preventing CS manifestations caused by persistent RNAPII stalling at DNA lesions and interruptions in transcription [10, 12]. Recent studies report that much bulkier DNA lesions, such as DNA‐protein crosslinks (DPCs), where RNAPII cannot access the damaged site, can also be handled by CSB [13, 14, 15]. Such DPCs, especially histone‐DPCs, are preprocessed by VCP/p97 and the proteasome, degrading them to remnant peptides before they can be removed by the TCR pathway [13]. The lack of CSA or CSB protein functions in CS patients causes severe neurological phenotypes. However, the specific endogenous DNA damage that induces transcription roadblocks and leads to CS has not been identified. DPCs are a likely candidate and are known to be induced by endogenous aldehydes, such as formaldehyde generated from one‐carbon metabolites like those produced during histone demethylation and one‐carbon metabolism [16]. This is supported by observations in a very rare genetic disorder known as AMeDS (aplastic anemia, mental retardation, and dwarfism syndrome), where due to the absence of enzymes for clearing endogenous aldehydes (encoded by *ADH5* and *ALDH2*) [13, 17, 18], patients exhibit CS‐like clinical features along with haematological abnormalities resembling those observed in Fanconi anemia (FA), a bone marrow failure syndrome [19]. TFIIS supports the RNAPII‐dependent cleavage of pre‐mRNA transcripts at cyclobutane pyrimidine dimer (CPD) lesions *in vitro*, thereby promoting resumption of RNA synthesis with the aid of CSB [20, 21]. However, in human cells, TFIIS‐mediated cleavage of RNA transcripts is indispensable only for the transcription restart of RNAPII from formaldehyde‐derived DPC lesions but not UV‐induced CPDs [13].

After the recognition and ubiquitination of stalled RNAPII by CSB/CSA, the ubiquitinated RPB1 recruits TFIIH, the DNA repair and transcription factor [22], with the help of UVSSA (UV‐stimulated scaffold protein A) [10]. UVSSA mutations have been identified in patients with UV‐sensitive syndrome (UV^S^S) [23, 24], who display acute sun sensitivity without any CS‐like devastating phenotypes [25], although cells derived from these patients show a complete absence of cellular TCR activity. The mild clinical features of UV^S^S may result from the prompt degradation of stalled RNAPII, preventing prolonged arrest at DNA lesions and ultimately signaling for apoptosis [24]. In the process of TFIIH recruitment, UVSSA interacts with the ubiquitin chain on RNAPII and the p62 subunit of TFIIH via the PH‐domain of p62 and the PH‐binding domain of UVSSA [10, 24, 26]. It also interacts with CSA and ELOF1, to support the complex formation [11]. USP7, which binds to UVSSA, inhibits the degradation of CSB and RNAPII by deubiquitinating excess ubiquitin chains [23, 27]. Afterward, UVSSA is ubiquitinated at the Lys414 residue, facilitating the transfer of TFIIH to RNAPII [10].

Subsequently, the helicases XPB and XPD in the TFIIH core complex unwind DNA around the lesion, with support from XPA and XPG. Loss of these XP proteins causes xeroderma pigmentosum (XP), a genodermatosis that predisposes individuals to skin cancer [28, 29]. Except for XP‐A (*XPA*‐defective), most XP patients do not exhibit a neurodegenerative phenotype; however, several cases with distinct mutations in *ERCC3/XPB* [30], *ERCC2/XPD* [31], or *ERCC5/XPG* [32] display the combined features of XP and CS (XPCS). In addition to these XPCS mutations, specific pathogenic variants in XPB, XPD, and the p8 subunit of TFIIH (TTDA, encoded by *GTF2H5*) can cause trichothiodystrophy (TTD) [33], which has similarities with CS but also includes sulphur‐deficient brittle hair [34]. XPCS and TTD are typically caused by TFIIH deficiency and may be distinct from CS‐A and CS‐B. During the loading of the nucleotide excision repair (NER) complex, stalled RNAPII is either backtracked or degraded, depending on the nature of DNA lesions, allowing for the recruitment of the NER incision endonucleases, ERCC1‐XPF (encoded by *ERCC4*) and XPG [35], to excise the DNA lesion. In exceptional cases, mutations in the ERCC1‐XPF endonuclease lead to the combined phenotypes of XP, CS, and FA (XPCSFA), because the shared 5′ damage incision machinery between NER and the inter‐strand crosslink (ICL) repair pathway, which is defective in FA [36]. After the removal of nucleotides containing damage, DNA polymerases and ligases eventually fill and seal the gap to complete the repair process [37, 38].

Taken together, TCR provides dual protection by activating DNA repair and processing DNA damage‐stalled RNAPII. This process triggers the assembly of the damage incision complex and facilitates the degradation of RNAPII, preventing prolonged stalling of transcription. Consequently, the fate of stalled RNAPII and maintaining precise transcription profiles are now recognised as crucial links between TCR and related human disorders, preventing transcription arrest and protecting against neurodegeneration.
